# Measuring implicit associations between food and body stimuli in anorexia nervosa: a Go/No-Go Association Task

**DOI:** 10.1007/s40519-023-01621-9

**Published:** 2023-11-02

**Authors:** Clara Lakritz, Sylvain Iceta, Philibert Duriez, Maxime Makdassi, Vincent Masetti, Olga Davidenko, Jérémie Lafraire

**Affiliations:** 1 Centre de Recherche de l’Institut Paul Bocuse, Ecully, France; 2 Université Paris-Saclay, AgroParisTech, INRAE, UMR PNCA, 91120 Palaiseau, France; 3 Département de Psychiatrie et de Neurosciences, Université Laval, Québec, QC Canada; 4 Centre de Recherche de l’Institut Universitaire de Cardiologie et de Pneumologie de Québec-Université Laval, Québec City, QC Canada; 5 Université Paris Cité, Institute of Psychiatry and Neuroscience of Paris (IPNP), INSERM U1266, 75014, Paris, France; 6 GHU-Paris Psychiatrie et Neurosciences, Hôpital Sainte Anne, 75014, Paris, France; 7 Clinea Psychiatrie France, Paris, France; 8 Laboratoire CHArt, Cognitions Humaine et ARTificielle,, EPHE - PSL, École Pratique des Hautes Études - Paris Sciences Lettres, Campus Condorcet, Aubervilliers, France

**Keywords:** Categorization, Food, Body image, Anorexia nervosa, Implicit association

## Abstract

**Purpose:**

The present study aimed to explore the implicit associations between food and bodily stimuli in patients with anorexia nervosa (AN) and control subjects (HC).

**Methods:**

A Go/No-Go Association Task was administrated to 55 participants (28 AN and 27 HC), using food stimuli (low-calorie food vs. high-calorie food) and body stimuli (underweight vs. overweight bodies).

**Results:**

We evidenced an implicit association between food and body stimuli in the AN group, whereas the HC group only showed a tendency. AN and HC groups also exhibited different categorization strategies: the AN group tended to categorize stimuli as low-calorie foods and underweight bodies less than the HC group, and they tended to categorize stimuli as high-calorie foods and overweight bodies more than the HC group.

**Conclusion:**

The present study revealed for the first time specificities of the AN population’s implicit association between food and body stimuli in terms of association strength and categorization strategy. Furthermore, the results suggest that combining implicit methodologies with other methods could contribute to a better characterization of the physiopathology of AN.

**Level of evidence:**

Level I, experimental study.

**Supplementary Information:**

The online version contains supplementary material available at 10.1007/s40519-023-01621-9.

## Introduction

Anorexia nervosa (AN) is one of the deadliest psychiatric illnesses [1, 2], characterized by severe restriction of energy intake relative to requirements, an intense fear of gaining weight, and a disturbance in the way in which one’s body weight or shape is experienced [3]. Two subtypes exist in AN: the binge-eating/purging subtype, which is characterized by regularly engaging in binge-eating or purging, and the restrictive subtype, which does not engage in these behaviors. For a decade, research on AN has shifted from studying overt behaviors to examining cognitive underpinnings that may contribute to the disorder's development, course, and treatment [4]. Among the set of cognitive factors potentially involved in the expression of AN, most research has focused on the reward system [5–7], executive functions [8–11], emotion regulation [12, 13], attentional bias [14, 15], and body perception [16, 17]. Body perception has been investigated the most, with research showing that patients suffering from AN tend to overevaluate shape and weight [16] and exhibit high body dissatisfaction [18]. Cognitive factors in AN have been investigated at both explicit and implicit levels. Implicit mechanisms are appropriate in populations that might exhibit self-presentation biases or social desirability biases, which is true of the population suffering from AN [19, 20]. These biases might lead, under certain circumstances, to a discrepancy between declarative data, which participants report directly (e.g., questionnaire responses), and data obtained using implicit methods, such as timing measurements to assess associations [21]. However, only a few studies have investigated how patients suffering from AN perceive and reason about food, while patients suffering from AN exhibit obsessive thought about eating [22].

Most research about food perception and reasoning in AN has focused on the reward system and has revealed a decreased preference for high-fat/calorie foods at explicit and implicit levels [23, 24]. Studies have also shown that anxiety traits and fear of gaining weight lead to food avoidance and limitation of caloric intake, specifically calories derived from fat [23, 25]. Studies on attentional bias, which is the tendency to focus on certain elements while ignoring others, toward food did not come to a consensus [23], even if recently Paslakis and colleagues [26] evidenced reduced implicit attentional bias toward food in AN in their systematic review. One study in social psychology conducted by Urdapilletta and colleagues [27] revealed that when patients suffering from AN classified food items, one criterion was their effect on the body, for example several items were grouped together because they were all considered difficult to eliminate, as they were too rich or too fatty and therefore indigestible. This literature suggests that patients with AN may perceive food based on its real or alleged effects on the body (regardless of the accuracy of these effects), and this is driven by the intense fear of gaining weight. However, no one has tested the association between the food and body domains, even though this association could be a distinctive feature of AN.

Methods that test the strength of associations between stimuli in distinct domains (e.g., between faces varying in ethnicity and moral predicates such as “good” and “bad”) are called implicit association methods. They have been used in AN to investigate body perception or food perception separately. Izquierdo and colleagues [28] investigated body perception with tests of the implicit associations between pro-dieting vs. non-dieting and true vs. false in a first task, and pictures of underweight vs. normal-weight models and positive vs. negative words in a second task. Results revealed pro-thin/anti-fat implicit biases in AN that were predictive of disordered eating and body image dissatisfaction, over and above the corresponding explicit biases. The same tasks helped Borgers and colleagues [29] to discover that the severity of symptoms in AN correlated with a higher implicit drive for thinness. Moreover, Smith and colleagues [30] used these methods to show stronger associations between emaciation and both beauty and ugliness. Concerning food perception, one study has examined the association between food and moral attributes in AN without finding any difference between the AN group and the healthy control subjects (HC) [31]. Another looked into the association between food and body image, specifically examining the relationship between meal portions and body size using an implicit association task [32]. However, it did not explore this link within the context of AN, but between restrained and unrestrained eaters. Contrary to what the authors expected, restrained and unrestrained eaters showed equally strong automatic associations between large meals and fat words, and between small meals and thin words.

Given the available evidence suggesting that food perception and body concerns are especially entangled in subjects with AN, the present study aimed at filling two gaps: a theoretical gap, as the strength of the relationship between food perception and body image has not been studied in AN, and a methodological gap, investigating the strength of this association using implicit association methods.

Based on the existing literature on the food and body variables that might trigger emotional responses or rejection in patients with AN, we tested the following hypotheses:

- (H1): all participants (HC and patients with AN) implicitly associate high-calorie foods with overweight bodies and low-calorie foods with underweight bodies;
- (H2): Patients with AN associate body stimuli with food stimuli more strongly than HC.

In addition, we explored the categorization strategies in subjects with AN compared to HC. Categorization strategies are response biases that avoid a particular type of error problematic for the subject [33]. For instance, if you must decide whether the thing you see is a snake or a stick in a forest, you might have the tendency to avoid thinking a snake is a stick, rather than mistaking a stick for a snake, due to the higher risk involved in the former. As seen in this example, risk influences categorization strategies. Uncertainty influences them also, as the tendency to avoid one type of error might be higher in a very uncertain situation. Indeed, in the same example, if the forest is in a deep fog, the tendency to avoid mistaking a snake for a stick might be higher than if you walk in the forest in a sunny day where you are able to clearly see each stick. Categorization strategy is worth exploring in the present study as patients with AN can experience both risk and uncertainty when classifying food and body stimuli according to calorie content and BMI.

## Methods

### Participants

No previous research investigated the strength of the implicit association between food and body stimuli in AN, so power analysis estimates was determined based on research that assessed implicit associations between meal sizes and body sizes among restrained and unrestrained eaters [32]. Following their results, a sample of 27 participants in each group (AN and HC) would be needed to obtain a similar effect size with a power of 80 and an alpha level of 0.05.

Sixty-nine women (28 with AN and 41 HC) completed this experiment. A first recruitment took place in June and July 2019 before the COVID-19 pandemic: 15 women with AN (33% binge/purging subtype; 67% restrictive subtype) and 25 HC completed the experiment. A second recruitment took place in December 2022 after the COVID-19 pandemic: 13 women with AN (40% binge/purging subtype; 60% restrictive subtype) and 16 HC completed the experiment. The participants in the two recruitments did not differ significantly in age or BMI (all p values > 0.05).

In the first recruitment, psychiatrists recruited the patients with AN. Patients with any severe comorbidity (e.g., major depressive disorders), with a BMI below 12, or having a prescription of benzodiazepine or an antipsychotic were excluded. All 15 patients were included in the analyses (mean age = 23.10, SD = 4.7, mean BMI = 16.70, SD = 1.5). One patient did not complete the age, height and weight, and questionnaire information but was included in the task analyses. Researchers recruited the HC through email databases of French universities and compensated them with a 10€ voucher. All HC group recruits were evaluated with psychometric scales to exclude participants with eating disorders or orthorexic characteristics. The HC completed the ORTO-15 [34] with the revised scoring suggested by Meule and colleagues [35] (Cronbach’s alpha (α) was 0.78) and the Eating Disorder Inventory II—Short Form (EDI-II-24, [36]) (α = 0.74), to assess, respectively, orthorexic and eating disorders traits. The HC who presented eating disorder symptoms (ORTO-15 < 35 or EDI > 52) were excluded from the analyses. Of 25 respondents, 15 were included in the HC group (mean age = 23.50, SD = 3.1, mean BMI = 22.00, SD BMI = 2.6).

In the second recruitment, psychiatrists recruited 13 patients with AN who were included in the analyses (mean age = 27.60, SD = 6.6, mean BMI = 16.10, SD = 1.4) using the same exclusion criteria. We refined the screening of the HC group on the basis of recent publications [37–39] and chose the following reliable questionnaires: the French version of the Eating Habits Questionnaire [37] to assess orthorexic traits (α = 0.93), and the French versions of the Eating Disorder Examination Questionnaire (EDEQ) [40] (α = 0.91) and the SCOFF questionnaire [41] (α = 0.83) to assess eating disorders traits. The HC who presented eating disorder symptoms (SCOFF > 2, after the 95th percentiles of EDE-Q and EHQ scores) were excluded from the analyses. Of 16 respondents, 12 were included in the HC group (mean age = 26.00, SD = 5.4, mean BMI = 18.64, SD BMI = 3.2).

With both recruitments, 55 participants were included in the analyses (mean age = 24.33, SD = 4.6, mean BMI = 19.06, SD BMI = 3.32). We found no difference between the participants recruited before and after the COVID-19 pandemic on age, BMI, satiety level, and the variables used, except on reaction times: we found an average increase of almost 73 ms for all the participants of the second recruitment. Because the results were compared between the AN and HC groups, this difference did not change the outcome of our results.

This study was performed in line with the principles of the Declaration of Helsinki. Approval was granted by the Ethics Committee of University Lyon 1 (2019/ ID-RCB Number: 2019-1A01595 52) and Paris (2015-A01194-45). Informed consent was obtained from all individual participants included in the study.

### Stimuli

The test stimuli were 40 color photographs, including 32 food items and 8 images of bodies (see Supplementary Materials Table 1 and OSF repository).

#### Food stimuli

Food stimuli were taken from the FoodPics database [42]. Stimuli were categorized in terms of their energy density (calories per 100 g and calories per image). We included 16 low-calorie stimuli (mean kcal/100 g = 47.6, SD = 25.6; mean kcal/image = 52.9, SD = 32.8) and 16 high-calorie stimuli (mean kcal/100 g = 355.3, SD = 184.3; mean kcal/image = 594.7, SD = 375.0). Because people perceive foods that are more processed to be more caloric [43], the degree of processing was controlled by including 8 perceived natural and 8 perceived processed foods in both the low- and high-calorie groups, following Blechert and colleagues’ classification within each category of energy density.

#### Body stimuli

We used a subset of 3D-graphics body stimuli taken from a larger database of computer-generated pictures of women’s bodies constituted by Moussally and colleagues [44]. Among the body items, we included 4 perceived to be underweight (BMI 13.2 to 19.6) and 4 perceived to be overweight (BMI 21.6 to 120.2). Among the body items, we included 4 perceived to be underweight (BMI 13.2 to 19.6) and 4 perceived to be overweight (BMI 21.6 to 120.2), according to weight categories described by the WHO [45]. A pre-test we conducted on 5 control participants indicated that the body stimuli were well discriminated.

### Go/No-Go Association Task

We administered a Go/No-Go Association Task (GNAT) [46] to the participants through the E-Prime 3.0 [47] and Labvanced [48] software. Participants were asked to press the space bar when they detected items that belonged to the target categories and to not press any key when presented items did not belong to the target categories. The GNAT is designed to measure the association between a target category and two poles of an attribute dimension (e.g., good/bad). In this GNAT, the two target categories are low-calorie food and high-calorie food and the two poles of the attribute correspond to underweight and overweight bodies. The GNAT included 4 practice single blocks and 4 combined blocks (Supplementary Materials Table 2). In the 4 practice blocks, participants learned to discriminate between two categories: low-calorie food from high-calorie food and underweight bodies from overweight bodies.

**Fig. 1:**
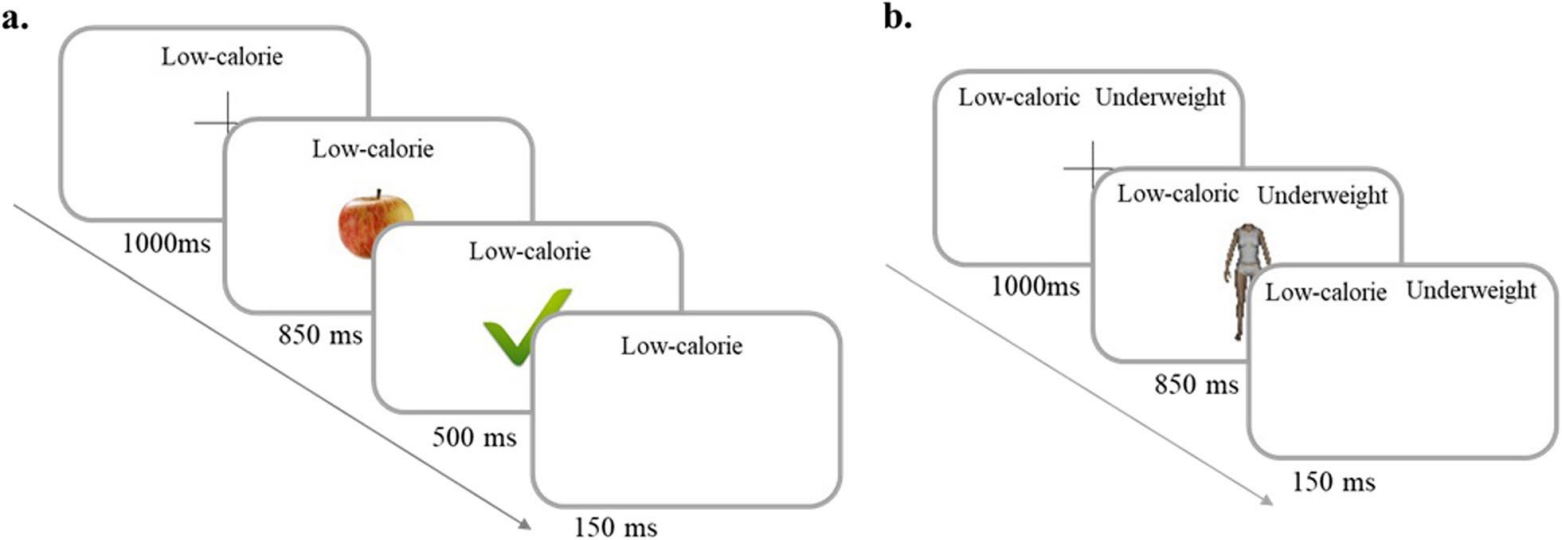
Stimulus sequences during practice block (**a**) and combined block (**b**). Interstimulus interval (ISI): 1 650 ms in practice blocks with feedbacks, ISI: 1 150 ms in combined blocks. For the purpose of this paper, this figure is an English translation of the task, whereas it was administrated in French

In each combined block, a target category (e.g., low-calorie food) was paired with an attribute (e.g., underweight body). In Block 1, participants had to press the bar if they saw a low-calorie food or an underweight body on the screen (target categories), and not press the bar if any other stimulus appeared on the screen (Block 1). Target categories for the second combined block were high-calorie food and overweight bodies (Block 2); they were low-calorie food and overweight bodies for the third one (Block 3); and they were high-calorie food and underweight bodies for the fourth one (Block 4). Among the 4 combined blocks, 2 represented the congruent conditions, in which the association between the target categories was hypothesized to be stronger (Blocks 1 and 2). For each block (practice or combined), distractor stimuli were the opposite of the target stimuli. Each stimulus was presented several times (2 times for food stimuli and 8 times for body stimuli). The stimulus sequences can be seen in Fig. 1.

### Procedure

We conducted the experiment in a quiet testing room. The participants’ level of satiety was evaluated on a 7-point Likert scale ranging from “not at all” to “extremely” hungry. The following instructions were given to the participants: “Press the space bar only for images belonging to the following categories (e.g., low-calorie food or underweight body). Otherwise, do nothing. Answer WITH AS FEW ERRORS AS POSSIBLE, and as quickly as possible.” We used capital letters to emphasize accuracy over speed as we wanted to favor analyses of errors based on previous work in the same population [31]. During the practice single blocks, participants were given a feedback after each trial: a green circle or a red cross corresponding to a correct or an incorrect response, respectively. The purpose of the practice single blocks was simply to accustom the participant to the task. No information on the rate of correct answers or the average response time was given to the participant at the end of each training block. The 4 combined blocks were presented in a random order across participants, without feedback.

Confirmation of stimulus choices: After the task, we asked participants to classify each body stimulus as underweight or overweight. In addition, in the second recruitment, we asked them to also classify each food stimulus as low-calorie or high-calorie. The stimuli were presented one by one in a randomly assigned order.

Finally, participants had to complete the questionnaires mentioned above. They had a total of one hour to complete the experiment and the questionnaires. Each individual session lasted approximately 35 min.

### Data recording and analyses

Individual response times (ms) and response were recorded. Analyses were conducted on the critical trials using Rstudio 3.6.0 R © software. The effects were considered significant when *p* < 0.05.

#### Confirmatory analyses

Reaction times (RT) and type of responses were analyzed. Data analyses assigned each block for each participant a score for hits (i.e., pressing the space bar when stimuli were in the target category) and a score for false alarms (i.e., when stimuli were distractors). Based on signal detection theory, we computed A’, an index of discriminability [49, 50]. The A’ index ranges from 0 to 1, with 0.5 indicating responses at chance level, and 1 indicating maximum discriminability. To test our hypothesis of an association between energy density and visual BMI (H1), we tested whether a facilitating effect on participants’ responses (shorter RT or higher A’) occurred in congruent conditions. To test our hypothesis of a difference of strength of association between our groups, we computed two indices, one with the RT and one with A’ (H2). Regarding RT, we computed the D-measure, which reflects the effect size and is conceptually similar to Cohen’s d [51]. D-measure was compared between groups with Student’s test. Regarding discriminability, we computed the difference in A’ between conditions and compared it between the AN and HC groups with a Mann–Whitney U test. Effect sizes were computed with the Cohen’s d formula [51]. An effect size of 0.2 to 0.5 is considered small, 0.5 to 0.8 is considered medium, and greater than 0.8 is considered large.

For the confirmation of stimulus choices, we calculated the percentage of errors made by each participant and each group and computed Chi-squared tests between groups.

Analyses were controlled for age, BMI, and satiety level.

#### Exploratory analyses

According to Signal Detection Theory [49, 50], another index can be computed from hit and false alarm rates: the participant’s decision criterion (β”), which is distinct from the discriminability index (A’). β” ranged from − 1 to + 1: − 1 indicates a liberal criterion, the participant exhibits a tendency to say that the signal is present;  + 1 indicates a conservative criterion, the participant exhibits a tendency to not say that the signal is present. In other words, the β” captures the *personal* response strategy in the presence of risk and/or uncertainty. In the present study, β” means were compared between groups in each condition and in each block to see if each group exhibited a specific strategy depending on the condition or block.

## Results

### Participants’ characteristics

Participants from the AN and HC groups did not differ in age or in state of satiety, but they differed in BMI, ORTO15 score, EDI-II-24 score, EHQ score, EDE-Q score, and SCOFF score (Supplementary Materials Table 3).

### Results of confirmatory analyses

Detailed results are available in Supplementary Materials, Tables 4 and 5. Mean RT were significantly different between conditions in the AN group {AN: *U*(28) = 232, *β* = 30.5, *p* = 0.016, 95% CI [8.27; 56.8]} with a moderate effect size of 0.68, whereas in the HC group, it was not significantly different {HC: *U*(27) = 254, *β* = 23.7, *p* = 0.113; 95% CI [− 1.03; 50.8]}. Discriminability was significantly different between conditions in the AN group {AN: *U*(28) = 542, *β* = − 0.022, *p* = 0.014, 95% CI [− 0.041; − 0.005]} with a moderate effect size of 0.78, whereas in the HC group, it was not significantly different {HC: *U*(27) = 449, *β* = − 0.013, *p* = 0.146; 95% CI [− 0.032; 0.005]}.

To evaluate whether the differences between incongruent and congruent conditions were different between the two groups, we looked at D-measures and A’. D-measures showed no significant difference {*t*(54) = 1.42, *β* = 9.01, *p* = 0.161, 95% CI [− 3.730; 21.800]} between the AN and HC groups, but A’ showed a significant difference {*U*(54) = 499, *β* = 0.013, *p* = 0.042, 95% CI [0.001; 0.029]} with a small effect size of 0.48.

Overall, the results showed a facilitating effect on participants’ responses (lower RT or higher A’) in the congruent condition compared to the incongruent condition in the AN group but not in the HC group, with a larger discriminability effect size in the AN group than in the HC group.

### Results of exploratory analyses

**Fig. 2:**
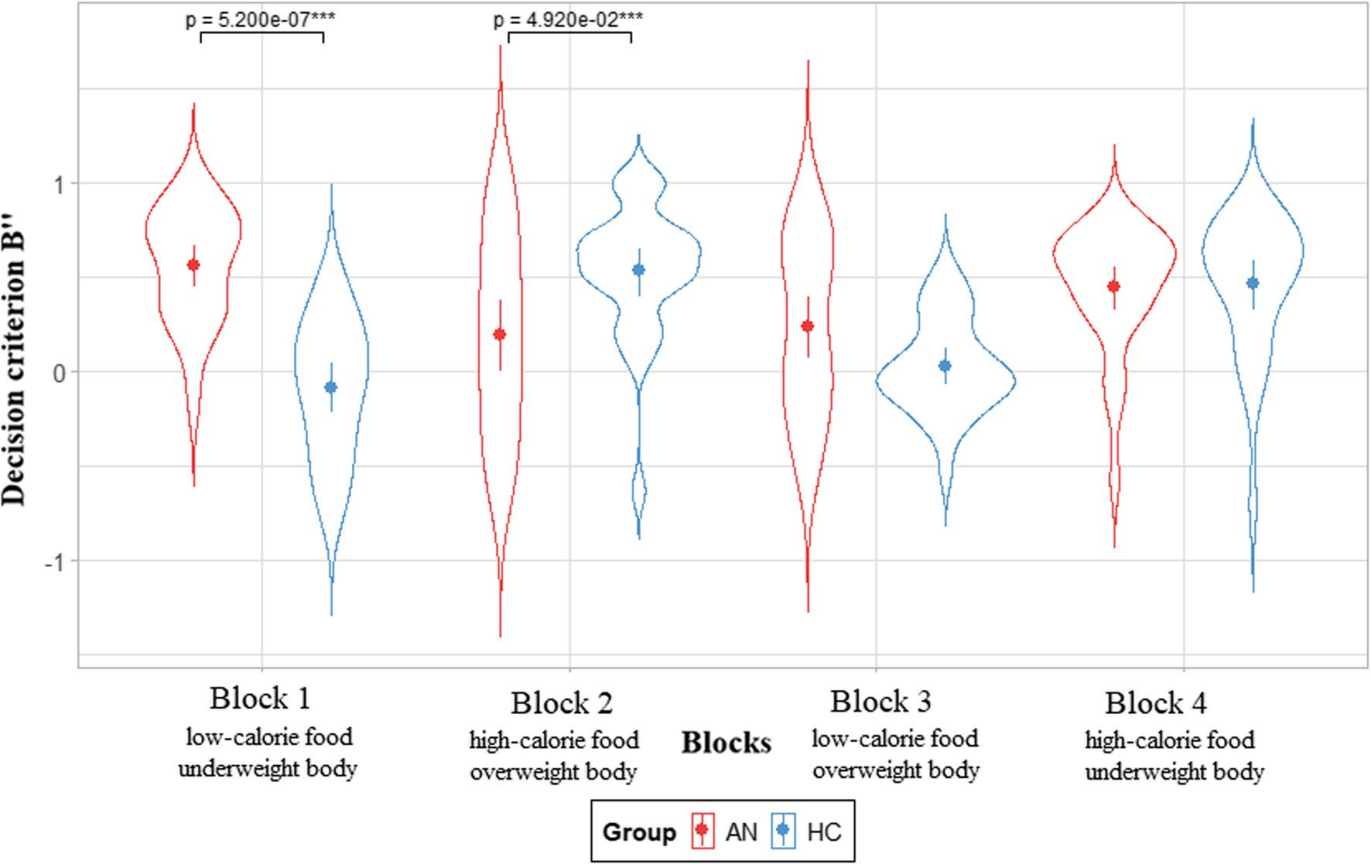
Decision criterion according to the groups and blocks. AN group = 28 patients with anorexia nervosa; HC group = 27 control participants; *p* = *p*-value of Mann–Whitney *U* test performed between groups; *** < 0.001

Taking each block separately, only the congruent blocks showed differences between AN and HC groups (see Fig. 2). Mean β” was positive and significantly higher in Block 1 (low-calorie food and underweight body as target categories) for the AN group than for the HC group {*U*(55) = 692, *β* = 0.642, *p* < 0.001, 95% CI [0.457; 0.830]} with a large effect size of 0.88. It indicated that the AN group’s decision strategy was more conservative in Block 1 (i.e., they tended to categorize fewer stimuli as low-calorie food and as underweight bodies than the HC group). On the contrary, in Block 2 (high-calorie food and overweight body as target categories), mean β” was significantly lower in the AN group than in the HC group {*U*(55) = 229, *β* = − 0.366, *p* = 0.012, 95% CI [− 0.632; − 0.080]} with a moderated effect size of 0.53. It indicated that the AN group was more liberal in Block 2 (i.e., they tended to categorize more stimuli as high-calorie food and as overweight bodies than the HC group). To summarize, the results revealed decision criterion differences between the AN group and the HC group in Blocks 1 and 2.

### Confirmation of stimulus choices

Regarding body stimuli, the AN group categorized significantly more underweight bodies as overweight than the HC group (AN: 36%, HC: 16%; *χ*^*2*^*(1,2)* = 9.28, *p* = 0.002, 95% CI [0.077; 0.338]). The difference between the two groups in terms of overweight bodies taken as underweight bodies was not significant (AN: 8%; HC: 15%; *χ*^*2*^*(1,2)* = 1.96, *p* = 0.162, 95% CI [− 0.182; 0.022]). Regarding food stimuli, the AN group categorized significantly more low-calorie food as high-calorie food (AN: 17%; HC: 8%; *χ*^*2*^*(1,2)* = 7.69, *p* = 0.006, 95% CI [0.009; 0.081]), and they categorized significantly less high-calorie food as low-calorie food than the HC group (AN: 9%; HC: 27%; *χ*^*2*^*(1,2)* = 18.2, *p* < 0.001, 95% CI [− 0.143; − 0.057]).

## Discussion

We studied the implicit association between food and body stimuli in a group of patients with AN compared to HC. We used energy density and visual BMI as main variables. To the best of our knowledge, this experiment is the first to assess the association between energy density and visual BMI in a population suffering from AN. Our data revealed the existence of this association in the AN group, without finding it in HC group. Findings also revealed that the AN group tended to avoid categorizing a high-calorie food for a low-calorie food, and to avoid categorizing an overweight body for an underweight body.

Results revealed the existence of the association between energy density and visual BMI in the AN group with moderate effect sizes, whereas we failed to find it in the HC group. Our finding emphasizes that the association investigated in this paper might be a distinctive feature of the population suffering from AN.

Our findings also shed light on a crucial aspect of AN that has often been overlooked: the heightened perception of risk associated with food choices. Our study demonstrated that individuals suffering from AN displayed a distinctive cognitive pattern (with moderate to large affect sizes compared to HC ), characterized by a strong inclination to avoid certain types of errors when categorizing food and body stimuli. They were more likely to avoid misclassifying high-calorie foods as low-calorie foods, which could conflict with their core objective of calorie restriction. They also were more likely to avoid misclassifying overweight bodies as underweight bodies, which is consistent with the intense fear of gaining weight in anorexia nervosa [3]. This heightened risk perception is consistent with previous research that highlighted anxiety-related tendencies in individuals with AN [52]. Indeed, White and colleagues [52] showed that anxious individuals were more likely to categorize threatening and neutral words as threatening than non-anxious individuals. It stands to reason that this elevated risk perception may contribute to the rigorous dietary restrictions observed in AN, as individuals strive to minimize any potential threat to their established eating patterns. Given the profound impact of anxiety on food-related decision-making, our results emphasize the importance of targeting anxiety-related interventions in AN. Such interventions could help individuals suffering from AN overcome the fear associated with food choices and develop healthier eating behaviors.

### Strengths and limits

This study measured, for the first time, implicit associations between body (visual BMI) and perceived energy density of food in people with AN. Results demonstrate that implicit methods can be used to detect AN and to explore the decision-making strategies of patients with AN in the presence of risk and uncertainty in the food and the body domains.

Several limitations of the present study need to be addressed in further research. First, the sample sizes were small and replication on larger sample sizes is needed to confirm the results. Moreover, a larger sample size could allow us to distinguish different AN subtypes, for example to distinguish participants with the restrictive type of AN from participants with binge-eating/purging AN.

In addition, the exclusion of people with orthorexia nervosa from the HC group did not allow us to explore this population and to contribute to the ongoing debate about the similarities and differences between orthorexia nervosa and other eating disorders [53]. Further research will have to include subjects with orthorexia nervosa who might exhibit body dissatisfaction and drive for thinness [54, 55].

### What was already known on this subject?

Patients with AN seem to exhibit a relationship to food driven by bodily concerns. Indeed, studies have shown that fear of gaining weight leads to food avoidance and limitation of caloric intake, and food categories were explained by bodily considerations among patients with AN. However, the strength of the association between body and food categories had not been clearly investigated in AN. This study aimed at filling this gap by measuring the strength of the associations between food and body stimuli within these populations, and by exploring their respective food and body categorization strategies.

### What does this study add?

Our study is the first to reveal implicit associations between food and body stimuli in participants with AN. As our data revealed specific patterns of the AN group, this opens the door for a better understanding of food perception at an implicit level in AN. In addition, results revealed heightened perceived risk in AN in the food and body domains. These findings demonstrate that implicit methods can be fruitfully used to measure the cognitive markers of AN and pave the way for further studies on the way food and bodily concerns and perceptions are intertwined in patients with AN. These results also give evidence for targeting anxiety-related interventions in the population suffering from AN to reduce perceived risk concerning food choices.

### Supplementary Information

Below is the link to the electronic supplementary material.

Supplementary file1 (DOCX 30 KB)

Supplementary file2 (DOCX 30 KB)

Supplementary file3 (DOCX 36 KB)

Supplementary file4 (DOCX 31 KB)

Supplementary file5 (DOCX 44 KB)

## Data Availability

The stimuli, datasets, and R code generated and analyzed are available in the OSF online repository at https://osf.io/tgxm4/?view_only=880dad413258489f8f368221504b4b3e and upon request from the corresponding author.
