# Supplementary material for: Measuring implicit associations between food and body stimuli in anorexia nervosa: a Go/No-Go Association Task
Source: Eat Weight Disord. 2023 Nov 2;28(1):93. doi: 10.1007/s40519-023-01621-9 (PMC10622378; doi:10.1007/s40519-023-01621-9)
Supplement: Supplementary file 1 — Supplementary file1 (DOCX 30 KB) [file 40519_2023_1621_MOESM1_ESM.docx]

Article title: Implicit Associations between food and silhouettes in anorexia nervosa

Authors: Clara Lakritz, Sylvain Iceta, Philibert Duriez, Maxime Makdassi, Vincent Masetti, Olga Davidenko, Jérémie Lafraire

Journal name: Eating and Weight Disorders – Studies on Anorexia, Bulimia and Obesity

Corresponding author: Jérémie Lafraire, Centre de Recherche de l’Institut Paul Bocuse, Ecully, France ; [jeremie.lafraire@institutpaulbocuse.com](mailto:jeremie.lafraire@institutpaulbocuse.com)

## Supplementary Materials Table 1

**SM Table 1** List of body stimuli from the database of Moussally and colleagues (2017).

| **Identification name** | **Stimulus BMI** | **BMI range** | **WHO Categories** | **Subscale set** |
| --- | --- | --- | --- | --- |
| UW15 | 15.67 | 15 – 15.99 | Severe underweight | Underweight |
| UW16 | 16.64 | 16 – 16.99 | Moderate underweight | Underweight |
| UW17 | 17.56 | 17 – 18.5 | Mild underweight | Underweight |
| UW19 | 19.61 | 18.5 – 24.9 | Normal | Underweight |
| OW21 | 21.55 | 18.5 – 24.9 | Normal | Overweight |
| OW27 | 27.37 | 25 – 29.9 | Overweight | Overweight |
| OW31 | 31.84 | 30 – 34.99 | Moderate obesity | Overweight |
| OW36 | 36.58 | 35 – 39.99 | Severe obesity | Overweight |

*Note*. WHO: World Wide Organization; BMI: Body Mass Index.
