# Supplementary material for: Measuring implicit associations between food and body stimuli in anorexia nervosa: a Go/No-Go Association Task
Source: Eat Weight Disord. 2023 Nov 2;28(1):93. doi: 10.1007/s40519-023-01621-9 (PMC10622378; doi:10.1007/s40519-023-01621-9)
Supplement: Supplementary file 2 — Supplementary file2 (DOCX 30 KB) [file 40519_2023_1621_MOESM2_ESM.docx]

Article title: Implicit Associations between food and silhouettes in anorexia nervosa

Authors: Clara Lakritz, Sylvain Iceta, Philibert Duriez, Maxime Makdassi, Vincent Masetti, Olga Davidenko, Jérémie Lafraire

Journal name: Eating and Weight Disorders – Studies on Anorexia, Bulimia and Obesity

Corresponding author: Jérémie Lafraire, Centre de Recherche de l’Institut Paul Bocuse, Ecully, France ; [jeremie.lafraire@institutpaulbocuse.com](mailto:jeremie.lafraire@institutpaulbocuse.com)

## Supplementary Materials Table 2

**SM Table 2** Summary of the design for the Go/No-Go Association Task (GNAT) blocks.

| N° of Block | Type of block | Task | Target categories | Number of trials |
| --- | --- | --- | --- | --- |
|  | Practice | Single task | Low-caloric food | 8 |
|  | Practice | Single task | Underweight silhouettes | 8 |
|  | Practice | Single task | High-caloric food | 8 |
|  | Practice | Single task | Overweight silhouettes | 8 |
|  | Practice | Combined task | Low-caloric food + Underweight silhouettes | 16 |
| 1 | Critical | Critical combined task | Low-caloric food + Underweight silhouettes | 128 |
|  | Practice | Combined task | High-caloric food + Overweight silhouettes | 16 |
| 2 | Critical | Critical combined task | High-caloric food + Overweight silhouettes | 128 |
|  | Practice | Combined task | Low-caloric food + Overweight silhouettes | 16 |
| 3 | Critical | Critical combined task | Low-caloric food + Overweight silhouettes | 128 |
|  | Practice | Combined task | High-caloric food + Underweight silhouettes | 16 |
| 4 | Critical | Critical combined task | High-caloric food + Underweight silhouettes | 128 |

## *Note.* Practice blocks were presented in a randomly assigned order between participants. Then, combined blocks were presented also in a randomly assigned order between participants. Only results of critical blocks were analyzed and presented in the results section. The number of the blocks in the present paper refers to the first column (N° of the blocks).
