# Supplementary material for: Measuring implicit associations between food and body stimuli in anorexia nervosa: a Go/No-Go Association Task
Source: Eat Weight Disord. 2023 Nov 2;28(1):93. doi: 10.1007/s40519-023-01621-9 (PMC10622378; doi:10.1007/s40519-023-01621-9)
Supplement: Supplementary file 3 — Supplementary file3 (DOCX 36 KB) [file 40519_2023_1621_MOESM3_ESM.docx]

Article title: Implicit Associations between food and silhouettes in anorexia nervosa

Authors: Clara Lakritz, Sylvain Iceta, Philibert Duriez, Maxime Makdassi, Vincent Masetti, Olga Davidenko, Jérémie Lafraire

Journal name: Eating and Weight Disorders – Studies on Anorexia, Bulimia and Obesity

Corresponding author: Jérémie Lafraire, Centre de Recherche de l’Institut Paul Bocuse, Ecully, France ; [jeremie.lafraire@institutpaulbocuse.com](mailto:jeremie.lafraire@institutpaulbocuse.com)

## Supplementary Materials Table 3

**SM Table 3** Participants’ characteristics (N=55) by group and comparison of scores between groups.

|  | Sample characteristics | | AN group | | | | HC group | | | | | *t* | | | | | *p* | | |
| --- | --- | --- | --- | --- | --- | --- | --- | --- | --- | --- | --- | --- | --- | --- | --- | --- | --- | --- | --- |
|  |  |  | M | | SD | | | M | | SD | | |  | |  | | |  |  |
|  | Whole sample | Age | | 25.1 | | 5.92 | | | 23.6 | | 2.69 | | | 1.18 | | .244 | | |  |
|  |  | BMI | | 16.5 | | 1.46 | | | 21.6 | | 2.52 | | | -9.23 | | < .001*** | | |  |
|  |  | Satiety level | | 1.59 | | 1.08 | | | 2.22 | | 1.53 | | | -1.75 | | .087 | | |  |
|  | 1^st^ recruitment (June 2019) | Age | | 23.1 | | 4.65 | | | 23.1 | | 2.80 | | | -0.05 | | 0.962 | | |  |
|  |  | BMI | | 16.7 | | 1.52 | | | 22.0 | | 2.66 | | | -6.70 | | < .001*** | | |  |
|  |  | EDI-II-24 | | 65.08 | | 16.50 | | | 39.43 | | 11.77 | | | 4.62 | | < .001*** | | |  |
|  |  | ORTO-15 | | 29.62 | | 6.14 | | | 37.79 | | 5.28 | | | -3.69 | | .001*** | | |  |
|  | 2^nd^ recruitment (December 2022) | Age | | 27.6 | | 6.56 | | | 24.2 | | 2.55 | | | 1.68 | | .228 | | |  |
|  |  | BMI | | 16.1 | | 1.37 | | | 21.2 | | 2.36 | | | -6.38 | | < .001*** | | |  |
|  |  | SCOFF | | 3.50 | | 1.78 | | | 1.88 | | 1.45 | | | 2.58 | | .018* | | |  |
|  |  | EDE-Q | | 4.04 | | 2.00 | | | 2.58 | | 1.56 | | | 2.39 | | .025* | | |  |
|  |  | EHQ | | 50.3 | | 7.58 | | | 35.3 | | 9.66 | | | 4.61 | | <.001*** | | |  |

*Note.* M = mean; SD = standard deviation; BMI, Body mass index; EDI-II, Eating Disorder Inventory – 24 items; t, test statistic for the comparison test of each variable between the two groups; p, pvalue of each test. * <.05, ** <.01, ***<.001
