# Supplementary material for: Measuring implicit associations between food and body stimuli in anorexia nervosa: a Go/No-Go Association Task
Source: Eat Weight Disord. 2023 Nov 2;28(1):93. doi: 10.1007/s40519-023-01621-9 (PMC10622378; doi:10.1007/s40519-023-01621-9)
Supplement: Supplementary file 4 — Supplementary file4 (DOCX 31 KB) [file 40519_2023_1621_MOESM4_ESM.docx]

Article title: Implicit Associations between food and silhouettes in anorexia nervosa

Authors: Clara Lakritz, Sylvain Iceta, Philibert Duriez, Maxime Makdassi, Vincent Masetti, Olga Davidenko, Jérémie Lafraire

Journal name: Eating and Weight Disorders – Studies on Anorexia, Bulimia and Obesity

Corresponding author: Jérémie Lafraire, Centre de Recherche de l’Institut Paul Bocuse, Ecully, France ; [jeremie.lafraire@institutpaulbocuse.com](mailto:jeremie.lafraire@institutpaulbocuse.com)

## Supplementary Materials Table 4

**SM Table 4** Mean and standard deviation of reaction times (ms) and discriminability indices by group and condition, with comparison between conditions.

|  | Condition | | | | | | | |  |  |
| --- | --- | --- | --- | --- | --- | --- | --- | --- | --- | --- |
|  | Congruent | | | | Incongruent | | | | *U or t* | *p* |
|  | M | | | SD | M | SD | | |  |  |
| Discriminability | 0.90 | | | 0.03 | 0.88 | | | 0.04 | *542* | *.014*** |
| RT (ms) | 538.49 | | | 49.21 | 564.63 | | | 50.66 | *-2.74* | *.004*** |
|  |  | | | |  | | | |  |  |
| For AN group only | (N = 28) |  | | |  | | | |  |  |
| Discriminability | 0.914 | | 0.02 | | 0.894 | | 0.03 | | *542* | *.014*** |
| RT (ms) | 555.65 | | 44.53 | | 586.21 | | 45.00 | | *-2.55* | *.013*** |
|  |  | |  | |  | |  | |  |  |
| For HC group only | (N = 27) |  | | |  | | | |  |  |
| Discriminability | 0.895 | | 0.04 | | 0.888 | | 0.03 | | *449* | *.146* |
| RT (ms) | 520.70 | | 48.22 | | 542.25 | | 46.97 | | *-1.66* | *.102* |

*Note.* M = mean; SD = standard deviation; U, test statistic for the Mann-Withney test of discriminability index between conditions; t, test statistic for the Student’s test of mean RT between conditions; p, pvalue of each test. ns = nonsignificant at α = .05; * p < .05; ** p < .01.
