# Supplementary material for: Measuring implicit associations between food and body stimuli in anorexia nervosa: a Go/No-Go Association Task
Source: Eat Weight Disord. 2023 Nov 2;28(1):93. doi: 10.1007/s40519-023-01621-9 (PMC10622378; doi:10.1007/s40519-023-01621-9)
Supplement: Supplementary file 5 — Supplementary file5 (DOCX 44 KB) [file 40519_2023_1621_MOESM5_ESM.docx]

Article title: Implicit Associations between food and silhouettes in anorexia nervosa

Authors: Clara Lakritz, Sylvain Iceta, Philibert Duriez, Maxime Makdassi, Vincent Masetti, Olga Davidenko, Jérémie Lafraire

Journal name: Eating and Weight Disorders – Studies on Anorexia, Bulimia and Obesity

Corresponding author: Jérémie Lafraire, Centre de Recherche de l’Institut Paul Bocuse, Ecully, France ; [jeremie.lafraire@institutpaulbocuse.com](mailto:jeremie.lafraire@institutpaulbocuse.com)

Supplementary Materials Table 5

**SM Table 5** Percentages of responses by group, block and type of responses

| Type of responses | | Hit | |  | False Alarm | | | Correct  Rejection | | Miss | | | A’ | | | β’’ | | |
| --- | --- | --- | --- | --- | --- | --- | --- | --- | --- | --- | --- | --- | --- | --- | --- | --- | --- | --- |
|  | | M | SD |  | M | SD |  | M | SD |  | M | SD |  | M | SD |  | M | SD |
| Congruent blocks | |  |  |  |  |  |  |  |  |  |  |  |  |  |  |  |  |  |
|  | Block 1 |  |  |  |  |  |  |  |  |  |  |  |  |  |  |  |  |  |
| AN | | 22.67% | 6.50 |  | 2.04% | 2.35 |  | 29.96% | 2.35 |  | 9.34% | 6.50 |  | 0.900 | 0.03 |  | 0.564 | 0.30 |
| HC | | 26.74% | 4.28 |  | 5.78% | 4.68 |  | 26.22% | 4.68 |  | 5.26% | 4.28 |  | 0.896 | 0.04 |  | -0.838 | 0.36 |
|  | Block 2 |  |  |  |  |  |  |  |  |  |  |  |  |  |  |  |  |  |
| AN | | 26.91% | 4.03 |  | 3.41% | 3.94 |  | 28.57% | 3.94 |  | 5.09% | 4.03 |  | 0.927 | 0.02 |  | 0.195 | 0.53 |
| HC | | 23.13% | 5.42 |  | 1.96% | 2.11 |  | 30.04% | 2.11 |  | 8.87% | 5.42 |  | 0.906 | 0.04 |  | 0.538 | 0.35 |
| Incongruent blocks | |  |  |  |  |  |  |  |  |  |  |  |  |  |  |  |  |  |
|  | Block 3 |  |  |  |  |  |  |  |  |  |  |  |  |  |  |  |  |  |
| AN | | 26.30% | 6.65 |  | 4.54% | 4.74 |  | 28.61% | 6.58 |  | 6.84% | 4.25 |  | 0.900 | 0.04 |  | 0.239 | 0.47 |
| HC | | 25.78% | 5.06 |  | 5.85% | 6.17 |  | 26.15% | 6.17 |  | 6.22% | 5.06 |  | 0.880 | 0.08 |  | 0.031 | 0.25 |
|  | Block 4 |  |  |  |  |  |  |  |  |  |  |  |  |  |  |  |  |  |
| AN | | 22.00% | 9.20 |  | 3.05% | 4.49 |  | 30.09% | 7.34 |  | 11.07% | 7.09 |  | 0.864 | 0.11 |  | 0.454 | 0.33 |
| HC | | 22.50% | 5.48 |  | 2.61% | 2.39 |  | 29.39% | 2.39 |  | 9.50% | 5.48 |  | 0.892 | 0.03 |  | 0.466 | 0.37 |

*Note.* M = mean; SD = standard deviation; AN group = 28 patients suffering from Anorexia Nervosa; HC group = 27 control subjects; A’ = discriminability index; β’’ = decision criterion
